# Eating frequency, timing of meals, and sleep duration before and after a randomized controlled weight loss trial for breast cancer survivors

**DOI:** 10.1007/s11764-024-01680-6

**Published:** 2024-09-24

**Authors:** Kelly D’cunha, Yikyung Park, Rebecca M. Leech, Melinda M. Protani, Louise Marquart-Wilson, Marina M. Reeves

**Affiliations:** 1 Faculty of Medicine, School of Public Health, The University of Queensland, Brisbane, QLD Australia; 2 Division of Public Health Sciences, Department of Surgery, Washington University School of Medicine, St Louis, MO USA; 3 Faculty of Health, Institute for Physical Activity and Nutrition (IPAN), Deakin University, Geelong, VIC Australia

**Keywords:** Meal timing, Sleep quality, Chrononutrition, Chronotype

## Abstract

**Purpose:**

To examine eating frequency, timing of meals, and sleep duration before and after a weight loss intervention for breast cancer survivors.

**Methods:**

Female breast cancer survivors (*n* = 159; 55 ± 9 years; 31.4 ± 5.0 kg/m^2^; stage I–III, median [IQR] 9.5 [5.5] months post-diagnosis) participated in a randomized controlled trial of a 12-month weight loss intervention versus usual care. Eating frequency, proportion of daily calories consumed after 5 PM, eating after 8 PM, nightly fasting duration, and sleep duration were estimated and categorized based on existing associations with factors influencing breast cancer prognosis and breast cancer outcomes. These behaviors at baseline were compared to women from an Australian national survey with similar age and BMI range. Mixed-effects linear regression models were used to examine the changes in health behaviors from baseline to 18 months between intervention and usual care groups.

**Results:**

Before the trial, eating after 8 PM (67%) was higher, and short nightly fasting duration (< 13 h, 83%) and long sleep duration (> 9 h/day, 26%) were marginally higher, in breast cancer survivors than women in the national survey (52%, 75%, and 17%, respectively). “Less optimal” eating behaviors and sleep duration tended to co-occur. Behaviors remained unchanged over the 18-month follow-up, irrespective of the study group (*p* > 0.05; Cohen’s effect sizes < 0.3).

**Conclusions:**

Later timing of eating and long sleep duration were prevalent in breast cancer survivors and continued following a weight loss intervention.

**Implications for Cancer Survivors:**

Future multi-behavior interventions in breast cancer survivors should consider specific messages to target eating timing behaviors and sleep.

**Supplementary Information:**

The online version contains supplementary material available at 10.1007/s11764-024-01680-6.

## Background

Breast cancer is the most diagnosed cancer globally [1]. However, with earlier diagnosis and advances in treatment, women are now surviving early-stage breast cancer and living longer [1]. Yet, most breast cancer survivors endure the detrimental long-term sequelae of treatment [2, 3] and are at increased risk of developing comorbid conditions (e.g., cardiovascular conditions), cancer recurrence, and a second primary cancer [4–7]. With growing populations of breast cancer survivors, a better understanding of the means to improve health and cancer outcomes remains a research priority.

Traditionally, research has focused on excess body weight, physical activity, and diet to improve survivorship after breast cancer [8]. However, more recently, eating frequency, timing of meals, and sleep parameters have been considered for the roles they play in breast cancer incidence and survivorship. This includes eating behaviors and sleep patterns that are misaligned with the phase of our internal body clocks (known as circadian misalignment), i.e., eating and staying awake during biological “night” or vice-versa [9]. A recent systematic review of eight studies summarizing the evidence on these behaviors post-diagnosis, reported that sleeping longer overnight (≥ 9 hours [h] versus 6–8 h) was consistently associated with an increased risk of all-cause mortality, and breast cancer mortality, recurrence, and progression, and indicators of better sleep quality were associated with a lower risk of all-cause mortality [10]. Additionally, seminal research in the review found that shorter nightly fasting duration (< 13 h versus ≥ 13 h) was associated with a higher risk of breast cancer recurrence [11]. Further, eating frequency, proportion of evening calories, eating after 8 PM, and nightly fasting duration have been associated with obesity and biomarkers of inflammation, factors known to adversely affect breast cancer prognosis [11, 12].

An individual’s affinity for activity and rest (known as chronotype) later in the day (late chronotype), compared to earlier (early chronotype), has also been associated with adverse health outcomes, including a higher risk of excess body weight [13], type 2 diabetes [14], and breast cancer [15]. Additionally, individuals with late chronotype, compared to early, sleep for shorter durations, experience poor sleep quality, consume unhealthier diets, and are more sedentary [16, 17].

Examining these emerging health behaviors in breast cancer survivors may inform the development of tailored and practical strategies to improve survivorship. Additionally, considering the associations of physical activity with sleep [18], and eating frequency with dietary intake and quality [19], we sought to examine if a traditional behavior-change weight loss intervention would elicit secondary benefits to emerging health behaviors. Therefore, this study aimed to examine eating frequency, timing of meals, and sleep parameters in breast cancer survivors, and the changes in these health behaviors in the context of a behavior-based, weight loss intervention trial.

## Methods

### Study design

This study reports findings from Living Well after Breast Cancer, a two-arm, parallel, randomized controlled trial registered with the Australian and New Zealand Clinical Trial Registry (ACTRN12612000997853). The trial aimed to evaluate the effectiveness of a 12-month, remotely delivered, weight loss intervention versus usual care in women following treatment for early-stage breast cancer. The trial protocol [20] and intervention effects on primary and key secondary outcomes have been previously reported [21, 22]. Ethics approval for the trial was received from the relevant hospital and university human research ethics committees [20].

### Study recruitment, participants, and randomization

The study recruited 159 women from seven hospitals in Brisbane (Australia) and the state-based cancer registry between October 2012 and December 2014 (Supplementary Fig. 1). Eligible participants included women aged 18–75 years, with a BMI of 25–45 kg/m^2^, who were diagnosed with early-stage breast cancer (stage I–III) within the previous two years and completed primary treatment (surgery, chemotherapy, or radiotherapy). Women were excluded if they were pregnant, not suited to unsupervised exercise or a weight loss program (e.g., unstable heart disease, taking pharmacological doses of warfarin, planning a knee or hip replacement in the next 6 months), and had greater than 5% weight loss in the past 6 months. Women were also excluded if they were unable to travel to Brisbane (study site) for data collection or had insufficient English to complete assessments and participate in the intervention.

Following the baseline assessment, women were randomized (1:1) into usual care or intervention groups using a computer-generated randomization sequence with uneven block sizes conducted by an off-site staff member unrelated to the study.

### Intervention

A detailed description of the intervention has been reported previously [20–22]. In summary, the intervention adopted a combined approach of improving diet quality, increasing physical activity, and behavioral therapy, with the aim to achieve 5% to 10% weight loss during the study period (12 months). The intervention was delivered remotely, entirely via telephone (phone calls and text messages) over the 12 months, following three phases—(1) an initial intensive 6-month phase (i.e., six weekly calls, 10 biweekly calls, and optional text messages); (2) followed by a 6-month extended care phase (i.e., six monthly calls and optional text messages); and (3) a final 6-month non-contact phase.

Participants allocated to the intervention group received a workbook, scale, measuring tape, pedometer, calorie-counter book, and self-monitoring diary. Additionally, after each data collection point, intervention participants received feedback comparing their dietary intake and physical activity levels with national and study recommendations [8, 23, 24], and a study newsletter from one of the national breast cancer organizations.

### Usual care

Participants allocated to the usual care group continued to receive standard medical care. Usual care participants also received the study newsletter and brief feedback on their dietary intake and physical activity levels after each data collection point; however, this was not compared with national and study recommendations.

### Data collection

Data were collected at baseline, 6 months, 12 months, and 18 months between November 2012 and October 2016, by staff blinded to the study group assignment. Retention was 89.9% at 6 months (*n* = 141), 81.8% at 12 months (*n* = 130), and 80.5% at 18 months (*n* = 128), with 78.0% of participants completing all four assessments (*n* = 124; Supplementary Fig. 1). Sociodemographic data (i.e., household income, highest education level received, employment status, marital status, ethnicity; Supplementary Table 1) and comorbidities (using the Charlson Comorbidity Index [25]) were self-reported at baseline via telephone interviews. Participant’s age and clinical characteristics (breast cancer diagnosis and treatment) were collected from cancer registry pathology records.

### Eating frequency, timing of meals, and sleep parameters

Definitions and categories of eating frequency, indicators of meal timing, and sleep parameters are detailed in Supplementary Table 2. The frequency and timing of meals (i.e., the proportion of daily calories consumed after 5 PM, eating after 8 PM, and nightly fasting duration) were collected from two unprompted 24-h dietary recall interviews (recalling one weekday and one weekend day) based on a 5-stage multi-pass method [26], conducted using FoodWorks Interview (Xyris Software, Brisbane, Australia). Eating frequency and timing of meals were estimated as the mean of the two dietary recalls and categorized based on existing associations with factors influencing breast cancer prognosis (e.g., obesity, biomarkers of inflammation) and breast cancer outcomes (Supplementary Table 2).

Sleep parameters considered for this study were sleep duration, sleep quality, and chronotype. Sleep duration and chronotype were estimated from self-reported sleep logs recorded consecutively for seven days (six nights). Sleep duration (in hours) was taken as the mean nightly sleep duration from sleep logs, weighted for potential differences between weekdays and weekends [27]. Chronotype was estimated for women who reported wake and sleep times for at least one weekday and weekend. Weekdays were considered from sleep time on Sunday to wake time on Friday, and weekends from sleep time on Friday to wake time on Sunday. Sleep quality (difficulty falling asleep/staying awake; perception of sleep quality) was estimated using single-item questions from the Insomnia Severity Index (ISI) included in the trial’s self-administered questionnaire [20, 28].

### Statistical analyses

Descriptive statistics were performed to report the prevalence of “less optimal” eating frequency and timing of meals, sleep duration, and indicators of sleep quality in our sample of breast cancer survivors at the study baseline. The descriptive statistics were reported in frequency and proportions (with 95% confidence intervals [CI] of the proportion). Eating frequency, timing of meals, and sleep duration were compared across subgroups (i.e., sociodemographic, clinical, and behavioral characteristics, and chronotype), using independent sample *t*-tests (where means ± SD are reported), Mann–Whitney tests (where median [IQR] is reported), and chi-squared tests (where *n* [%] are reported).

Where available, eating frequency, timing of meals, and sleep duration were compared to women from an Australian national sample with similar age and BMI range from the National Nutrition and Physical Activity Survey (NNPAS) [29]. Details on the methods for assessing eating behaviors and sleep duration in this national survey are included in Supplementary Table 2. All percentage estimates from NNPAS data account for person weightings and survey design [29]. Proportions of those practicing “less optimal” health behaviors were compared between breast cancer survivors and women from the national survey descriptively, with differences greater than or equal to 10% (absolute) considered noteworthy. We also conducted this comparison stratified by age (mean age of sample: < 55 years versus ≥ 55 years). Comparable nationally representative data for sleep quality were not available and therefore not included in this comparison.

Linear mixed-effects models were used to examine changes within groups and intervention effects (relative to the control group) on eating frequency and timing of meals (i.e., proportion of daily calories consumed after 5 PM, nightly fasting duration) and sleep duration over the study follow-up period from baseline to 18 months. The models included fixed effects for the treatment arm, timepoint, and their interaction, and a random intercept for participants. Marginal means evaluated at mean values were used to report within-group changes over time and between-group differences. As per protocol [20], no potentially confounding variables (i.e., stage of disease, menopausal status, treatment, pre-diagnosis weight, employment status, and educational level) met the a priori criteria (i.e., statistically significant between-group differences for potentially influential confounding variables) for inclusion and were therefore not included in the models. However, given the meaningful differences in menopausal status between study groups (Supplementary Table 1), we ran additional analyses to adjust for menopausal status, with no significant changes in the estimates observed (results not shown).

The study sample size was powered a priori based on weight loss, the primary outcome of the trial, and was not powered for the outcomes of interest of the present study [20]. Therefore, Cohen’s *d* [30] effect sizes were calculated to assist with the interpretation of intervention effect sizes, with d (absolute) > 0.3 considered meaningful. All statistical analyses were performed using STATA (version 17, StataCorp, Texas). Statistical significance was set at *p* < 0.05.

## Results

At baseline, a total of 159 women were included in the trial (Supplementary Fig. 1). The women were, on average, 55 years old (± 9), with a BMI of 31.4 kg/m^2^ (± 5.0), and approximately 9.5 months (median [IQR] 5.5) post-diagnosis of early-stage breast cancer. Women in the intervention and usual care groups did not differ significantly by their sociodemographic and clinical characteristics except for noteworthy (≥ 10% difference) tendencies for women in the intervention group (compared to usual care) to be postmenopausal and have multi-comorbidities (Supplementary Table 1).

### Eating frequency and timing of meals

**Fig. 1:**
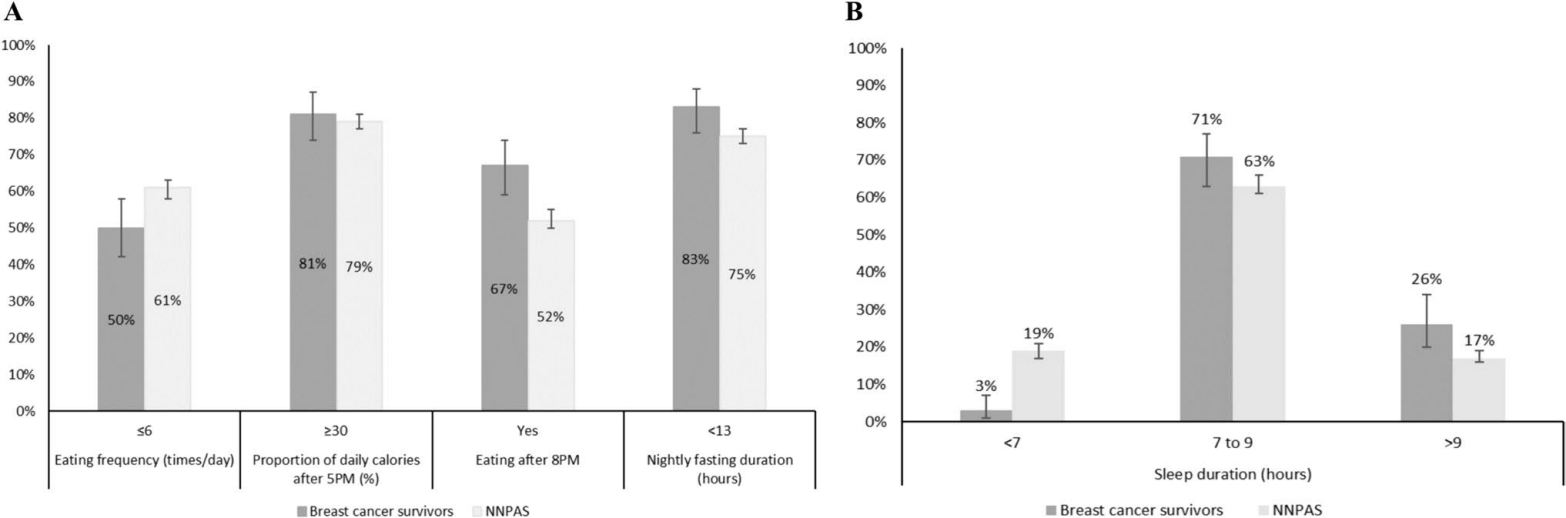
Proportion of breast cancer survivors from the Living Well after Breast Cancer trial (*n* = 159) engaging in “less optimal” eating behaviors (**A**), and sleep duration (**B**), compared to women from a national survey (NNPAS; *n* = 1735). The error lines represent the 95% confidence intervals calculated from the standard error of the proportions. Abbreviations: NNPAS, National Nutrition and Physical Activity Survey

At the study baseline, indicators of “less optimal” eating timing were highly prevalent in our sample of breast cancer survivors (Fig. 1). Eating late at night (after 8 PM) was higher and fasting for less than 13 h overnight was marginally higher in breast cancer survivors (67% and 83%, respectively) than in women in the NNPAS national survey (52% and 75%, respectively). When stratified by age (< 55 years versus ≥ 55 years; Supplementary Table 3), differences between breast cancer survivors and the NNPAS national survey were more notable in the younger age group (< 55 years) for eating after 8 PM only (77% versus 55%). Generally, breast cancer survivors who received higher education, were employed, had a higher household income, and were married tended to practice one or more “less optimal” eating timing behaviors (Supplementary Table 4). No associations were observed for differences in eating frequency and timing of meals by clinical characteristics, except for chemotherapy, whereby women who received chemotherapy tended to eat more frequently.

### Sleep parameters

The majority of breast cancer survivors (71%) slept between the recommended sleep duration of 7 to 9 h per night, while approximately one in four women (26%) reported oversleeping (> 9 h) (Fig. 1). When compared to women from the NNPAS national survey, a higher proportion of breast cancer survivors reported long sleep ([> 9 h]; 26% versus 17%), and fewer reported short sleep duration ([< 6 h]; 3% versus 19%). When stratified by age (Supplementary Table 3), the difference in long sleep duration between breast cancer survivors and the NNPAS national survey was observed only in the younger age group only (< 55 years; 27% versus 15%). Sleep duration did not differ by any other sociodemographic or clinical characteristics (Supplementary Table 4). Additionally, over a third of breast cancer survivors reported experiences of indicators of poor sleep quality (Supplementary Table 5).

### Co-occurrence of behaviors

The “less optimal” health behaviors tended to co-occur in breast cancer survivors (Table 1). Women who ate > 6 times per day (versus ≤ 6 times/day) and those who ate after 8 PM (versus not) had higher total energy intake. Women who fasted for a shorter duration overnight (< 13 h) tended to eat more frequently (> 6 times/day) and eat after 8 PM (versus not), compared to women who fasted for a longer duration overnight (≥ 13 h), while women who ate after 8 PM (versus not) tended to consume ≥ 30% of daily calories after 5 PM and were less likely to report problems falling asleep or waking up too early (Table 1 and Supplementary Table 6).

**Table 1:** Co-occurrence of eating frequency, timing of meals, and sleep duration in breast cancer survivors from the Living Well after Breast Cancer trial

Characteristics	Eating frequency (times/day)		*p*	Proportion of calories after 5 PM (%)		*p*	Eating after 8 PM		*p*	Nightly fasting duration (hours)		*p*	Sleep duration (hours)		*p*^a^
	> 6 (*n* = 80)	≤ 6 (*n* = 79)		< 30 (*n* = 30)	≥ 30 (*n* = 129)		No (*n* = 52)	Yes (*n* = 107)		≥ 13 (*n* = 27)	< 13 (*n* = 132)		≥ 7 to ≤ 9 (*n* = 112)	> 9 (*n* = 42)	
Energy (MJ) – mean ± SD	7.8 ± 2.1	6.3 ± 1.7	** < 0.001**	6.4 ± 1.8	7.2 ± 2.1	0.054	6.4 ± 1.8	7.3 ± 2.1	**0.008**	6.4 ± 2.0	7.2 ± 2.1	0.09	7.2 ± 2.2	6.6 ± 1.8	0.11
	*n* (%)														
Eating frequency (times/day)			-			0.66			0.08			** < 0.001**			**0.048**
> 6	-	-	-	14 (47)	66 (51)		21 (40)	59 (55)		5 (18)	75 (57)		60 (54)	15 (36)	
≤ 6	-	-	-	16 (53)	63 (49)		31 (60)	48 (45)		22 (81)	57 (43)		52 (46)	27 (64)	
Proportion of daily calories after 5 PM (%)			-			-			**0.007**			0.30			0.71
< 30	-	-	-	-	-	-	16 (31)	14 (13)		7 (26)	23 (17)		21 (19)	9 (21)	
≥ 30			-			-	36 (69)	93 (87)		20 (74)	109 (83)		91 (81)	33 (79)	
Eat after 8 PM	-	-	-	-	-	-			-			** < 0.001**			0.14
No	-	-	-	-	-	-	-	-	-	18 (67)	34 (26)		34 (30)	18 (43)	
Yes	-	-	-	-	-	-	-	-	-	9 (33)	98 (74)		78 (70)	24 (57)	
Nightly fasting duration (hours)			-			-			-			-			** < 0.001**
≥ 13	-	-	-	-	-	-	-	-	-	-	-	-	11 (10)	16 (38)	
< 13	-	-	-	-	-	-	-	-	-	-	-	-	101 (90)	26 (62)	

Descriptive statistics were performed to report on the co-occurrence of eating frequency, timing of meals, and sleep duration in breast cancer survivors at the study baseline, with differences between groups estimated using independent samples *t*-tests (where mean ± SD are presented) and chi-squared tests (where *n* [%] are presented). ^a^*p*-value excludes sleep duration < 7 h due to small sample size

Women who slept longer overnight (> 9 h) more commonly ate less frequently (≤ 6 times/day), and less commonly reported fasting for a short duration overnight (< 13 h), than those who slept within the recommended hours (7–9 h; Table 1). Longer overnight sleep (versus 7–9 h) was more commonly associated with “moderate” to “very severe” problems and difficulties falling asleep and staying asleep (Supplementary Table 6).

Breast cancer survivors with late chronotype, compared to early, tended to consume a greater proportion of daily calories after 5 PM (*p* < 0.05), eat after 8 PM (*p* < 0.05), and eat less frequently (≥ 10% difference) (Table 2).

**Table 2:** Eating frequency, timing of meals, and sleep duration by chronotype in breast cancer survivors from the Living Well after Breast Cancer trial (*n* = 156)

	Chronotype^a^			*p*
Eating and sleep behaviors	Early (*n* = 52)	Intermediate (*n* = 53)	Late (*n* = 51)	
Eating frequency (times/day)				
> 6	32 (62)	25 (48)	23 (44)	0.18
≤ 6	20 (38)	27 (52)	29 (56)	
Proportion of calories after 5 PM (%)				**0.022**
< 30	16 (31)	9 (17)	5 (10)	
≥ 30	36 (69)	43 (83)	47 (90)	
Eating after 8 PM				** < 0.001**
No	28 (54)	16 (31)	7 (13)	
Yes	24 (46)	36 (69)	45 (86)	
Nightly fasting duration (hours)				0.24
< 13	41 (79)	47 (90)	42 (81)	
≥ 13	11 (21)	5 (10)	10 (19)	
Sleep duration (hours)				0.12^b^
7 to 9	31 (65)	43 (83)	37 (73)	
> 9	17 (35)	9 (17)	14 (27)	

Data are number and percentage of participants *n* (%). ^a^*n* = 3 participants did not report sleep on either a weekend or weekday. ^b^*p*-value excludes sleep duration < 7 h due to small sample size. Descriptive statistics were performed to report on the associations between eating frequency, timing of meals, and sleep duration with chronotype in breast cancer survivors at the study baseline using chi-squared tests

### Changes in eating frequency and timing, and sleep duration after a weight loss intervention

No statistically significant or meaningful (*d* < 0.3) intervention effects (intervention minus usual care) were observed for eating frequency, nightly fasting duration, and sleep duration over the 18-month follow-up period (Table 3). Meaningful differences (*d* = 0.32) were observed for the proportion of daily calories consumed after 5 PM at the end of the intervention only (i.e., 12 months), favoring the usual care group.

**Table 3:** Intervention effects on eating frequency, proportion of daily calories consumed after 5 PM, nightly fasting duration, and sleep duration following a 12-month behavior change weight loss intervention for breast cancer survivors from the Living Well after Breast Cancer trial

Outcome	Timepoint	Intervention		Usual care		Intervention effect (intervention – usual care)		
		*n*	Mean change^a^ (95% CI)	*n*	Mean change^a^ (95% CI)	Mean difference (95% CI)	*p*^b^	*d*^c^
Eating frequency (times/day)	Baseline	79	6.37 ± 1.48	80	6.69 ± 1.85			
	6 months	73	0.20 (− 0.13, 0.53)	69	− 0.19 (− 0.53, 0.14)	0.07 (− 0.45, 0.60)	0.78	0.06
	12 months^d^	70	− 0.09 (− 0.44, 0.25)	60	− 0.38 (− 0.75, -0.02)	− 0.03 (− 0.57, 0.51)	0.91	0.03
	18-months	67	− 0.15 (− 0.51, 0.21)	61	− 0.34 (− 0.72, 0.03)	− 0.12 (− 0.67, 0.42)	0.66	< 0.001
Proportion of daily calories consumed after 5 PM (%)	Baseline	79	41.69 ± 12.48	80	41.69 ± 17.29			
	6 months	73	1.51 (− 1.66, 4.68)	69	− 0.09 (− 3.30, 3.11)	1.49 (− 2.36, 5.32)	0.45	0.12
	12 months^d^	70	0.44 (− 2.95, 3.84)	60	− 3.38 (− 6.91, 0.14)	3.72 (− 0.16, 7.60)	0.06	**0.32**
	18 months	67	− 2.03 (− 5.79, 1.72)	61	− 2.50 (− 6.31, 1.31)	0.35 (− 3.67, 4.38)	0.86	0.04
Nightly fasting duration (hours)	Baseline	79	11.86 ± 1.30	80	11.48 ± 1.32			
	6 months	73	− 0.26 (− 0.64, 0.12)	69	0.06 (− 0.32, 0.45)	0.05 (− 0.44, 0.55)	0.83	0.06
	12 months^d^	70	− 0.19 (-0.59, 0.19)	60	− 0.21 (− 0.62, 0.21)	0.38 (− 0.16, 0.93)	0.16	0.18
	18 months	67	− 0.30 (− 0.71, 0.09)	61	0.02 (− 0.40, 0.44)	0.05 (− 0.53, 0.63)	0.86	< 0.001
Sleep duration (hours)	Baseline	79	8.53 ± 0.91	80	8.54 ± 0.89			
	6 months	71	0.12 (-0.14, 0.37)	69	0.26 (0.01, 0.52)	− 0.14 (− 0.49, 0.21)	0.43	− 0.16
	12 months^d^	68	− 0.15 (− 0.41, 0.11)	59	− 0.05 (− 0.32, 0.22)	− 0.10 (− 0.46, 0.27)	0.60	− 0.04
	18 months	65	− 0.12 (− 0.39, 0.14)	59	0.11 (− 0.16, 0.38)	− 0.23 (− 0.59, 0.14)	0.23	− 0.18

Baseline values are represented as mean ± SD. ^a^Mean change from baseline values. ^b^*p*-value comparing change at each time-point between the study groups. ^c^Standardized effect (Cohen’s *d*): mean intervention effect divided by pooled baseline standard deviation of the outcome. ^d^End-of-intervention contact; primary endpoint

## Discussion

Eating frequency, timing of meals, and sleep duration are associated with breast cancer incidence and outcomes [11, 12, 31]. In this study, we observed that a higher proportion of breast cancer survivors ate late at night (after 8 PM) and fasted for a shorter duration overnight (< 13 h) compared to a nationally representative sample of women. Additionally, over a third of breast cancer survivors reported at least one indicator of poor sleep quality and while the majority slept within the recommended duration, a higher proportion of breast cancer survivors than the national sample, slept longer than 9 h per night. These behaviors did not change over the 18-month follow-up period, including in those who received the behavior-based weight loss intervention. Our findings indicate that in addition to traditional health behaviors, timing of meals and sleep may be important to target in women after a breast cancer diagnosis. Support to modify these behaviors may offer simple, practical, non-pharmacological means to improve survivorship, however, more targeted, and tailored interventions and messages are likely needed to modify them.

To the best of our knowledge, this is the first study to report on eating frequency and timing of meals in breast cancer survivors compared to the general population. Both breast cancer survivors and women from the national survey reported a high prevalence of “less optimal” eating timing behaviors, with a higher proportion of breast cancer survivors eating late at night and fasting for shorter duration overnight. The practice of these “less optimal” behaviors is likely a reflection of the environment we live in, where food is available and accessible around the clock, as well as time pressures due to family and work commitments [32], particularly for younger women as seen in this study. The high prevalence of these health behaviors is concerning for both women with and without breast cancer, given the associations of eating frequency and timing with breast cancer risk factors and prognosis [11, 12, 31].

While estimates of poor sleep quality or sleep disturbances are frequently described, at present there are limited reports on the prevalence of short or long sleep duration in breast cancer survivors [33, 34]. The majority of breast cancer survivors (71%) adhered to sleep recommendations (7–9 h); however, approximately one in four women reported sleeping longer than 9 h per night, slightly more than the general population, with this difference predominantly seen in younger breast cancer survivors (< 55 years). Given the consistent evidence demonstrating associations between long sleep duration and an increased risk of breast cancer progression and poorer survival [10], this finding is concerning. Of note, those who reported long sleep duration in this study, more commonly reported moderate to severe indicators of poor sleep quality (i.e., problems falling asleep and problems staying asleep). Thus, it is plausible that not all of those classified as “long sleepers” slept > 9 h but instead had interrupted or disrupted sleep. Previous studies of primarily breast cancer cohorts, have reported short sleep duration (≤ 6 or < 7 h) as more common (22–40%) [33, 34], with only ~ 12% of breast cancer survivors reporting long sleep duration (≥ 8 h) at ~ 6 months post-diagnosis [33]. Differences in prevalence estimates may reflect differences in time since diagnosis and sleep disturbances associated with breast cancer treatments [33, 34] and/or, more likely, measurement differences. Single-item questions recalling typical sleep duration (used in previous studies) have been shown to elicit shorter sleep duration compared to sleep logs [35].

Sleep disturbances are commonly reported in breast cancer survivors, with pooled estimates suggesting that 40% of women, at least 1 year post-diagnosis of breast cancer, experience sleep disturbances following treatment completion [36]. In this study, we found comparable levels with poor sleep quality/sleep disturbances ranging from 36 to 54%, and a third of survivors reporting overall dissatisfaction with their sleep patterns. Chemotherapy and experiences of menopausal symptoms (consequent to treatment) reportedly contribute to poor sleep quality in breast cancer survivors [34, 37]. Except for age, sleep duration was not associated with any sociodemographic or clinical characteristics, nor were indicators of sleep quality. However, menopausal symptoms were associated with sleep satisfaction (results not shown).

While indicators of sleep quality were mostly not associated with eating timing behaviors, sleep duration was, suggesting that the interaction or co-occurrence of eating and sleep timing behaviors are likely important for health and well-being of breast cancer survivors. Traditional health behaviors also tend to co-occur, and the evidence demonstrates that targeting multiple health behaviors is effective in managing chronic disease conditions [38].

Individuals’ chronotype, their natural inclination to sleep and be active at certain times [39], has also been associated with distinct eating timing and dietary behaviors and health profiles [40]. We found that breast cancer survivors with late chronotype (compared to early) more commonly ate late at night and tended to eat a greater proportion of their daily calories in the evening, consistent with previous studies [13, 40, 41]. People with evening/late chronotype are more likely to have a higher BMI, blood glucose, glycated hemoglobin, LDL cholesterol, and triglycerides levels and an increased risk of diabetes and cancer, compared to morning/early chronotype [13, 42]. While chronotypes itself may not be easily modifiable, understanding behaviors typically occurring in those with late chronotype may help to provide targeted advice and recommendations to improve behavior change and health outcomes.

Breast cancer survivors in this study were participants in a weight loss intervention trial which aimed to target traditional dietary and physical activity behaviors to achieve modest weight loss. Given the interplay between eating frequency and sleep and the behaviors targeted as part of the intervention, we sought to understand whether eating frequency, timing of meals, and sleep duration were modified over the 18-month follow-up period. However, we did not observe significant or meaningful changes in these behaviors. Previous trials of physical activity and diet in breast cancer and non-breast cancer populations have reported beneficial effects of the intervention on objectively measured sleep disturbance/quality [43–45]; however, evidence on the intervention effects on sleep duration is few, and yet to be examined for eating frequency and timing of meals. Our intervention did not specifically address these behaviors. Participants were advised on the benefits of exercise on sleep quality and were guided to have small, regular meals. With the increasing popularity of intermittent fasting at the time of the trial [46], on request by intervention participants, they were supported to safely follow specific time-restricted diets like intermittent fasting. Despite this, we did not observe any improvements in eating frequency and timing of meals, or sleep duration following the intervention. To achieve improvements, more specific, targeted, and tailored messaging on how to modify these health behaviors is likely needed.

Our study adds to the limited evidence to date on the prevalence and changes in eating frequency, timing of meals, and sleep duration, in breast cancer survivors. However, the study sample included predominantly White Caucasian women, limiting generalizability to other populations, especially given that sleep and eating practices often differ between ethnic and cultural backgrounds [47–49]. Although measures of sleep (duration and quality) were estimated via validated subjective tools (i.e., sleep logs and single-item questions), they may be less accurate than objective measures and prone to measurement errors and social desirability biases. Future studies should use objective measures, such as wrist-worn activity monitors, that are suitable and comfortable to wear overnight, to ensure accurate measurement and categorization of sleep [50]. Additional data on eating and sleep environments can provide a holistic understanding of enablers and barriers to engaging in desirable behaviors, which may prompt targets for more tailored interventions. Finally, given the high prevalence and co-occurrence of “less optimal” eating and sleep behaviors in breast cancer survivors, further research investigating the impact of these behaviors on intermediate outcomes (like biomarkers) and hard endpoints (like survival) are warranted.

In conclusion, our findings of a higher prevalence of eating late at night (after 8 PM), shorter overnight fasting duration, and long sleep duration in breast cancer survivors are particularly concerning given the associations between these health behaviors and breast cancer prognosis. These behaviors have not specifically been the focus of recommendations for cancer survivors to date [51]. To improve not only breast cancer prognosis but overall health of breast cancer survivors, future studies are recommended to consider multi-behavior interventions with more targeted messaging to modify these health behaviors.

## Supplementary Information

Below is the link to the electronic supplementary material.

Supplementary file1 (PDF 492 KB)

## Data Availability

The datasets generated during and/or analysed during the current study are available from the corresponding author on reasonable request and ethics approval.
