# Supplementary material for: Eating frequency, timing of meals, and sleep duration before and after a randomized controlled weight loss trial for breast cancer survivors
Source: J Cancer Surviv. 2024 Sep 24;20(2):704–14. doi: 10.1007/s11764-024-01680-6 (PMC12988965; doi:10.1007/s11764-024-01680-6)
Supplement: Supplementary file 1 — Supplementary file1 (PDF 492 KB) [file 11764_2024_1680_MOESM1_ESM.pdf]

Eating frequency, timing of meals, and sleep duration before and after a randomised controlled weight loss trial for breast cancer survivors.

Journal of Cancer Survivorship

Kelly D'cunha[1], Yikyung Park[2], Rebecca M. Leech[3], Melinda M. Protani[1], Louise Marquart-Wilson[1], Marina M. Reeves[1,*]

[1]*Faculty of Medicine, School of Public Health, The University of Queensland, Brisbane, QLD, Australia*

[2]*Division of Public Health Sciences, Department of Surgery, Washington University School of Medicine, St Louis, MO, USA*

[3]*Faculty of Health, Institute for Physical Activity and Nutrition (IPAN), Deakin University, Geelong, VIC, Australia*

***Correspondence:**

Marina M. Reeves, PhD, Adv APD, School of Public Health, Faculty of Medicine, The University of Queensland, Level 4, Public Health Building, 288 Herston Rd, Brisbane, Australia 4006; marina.reeves@uq.edu.au.

Supplementary Figure

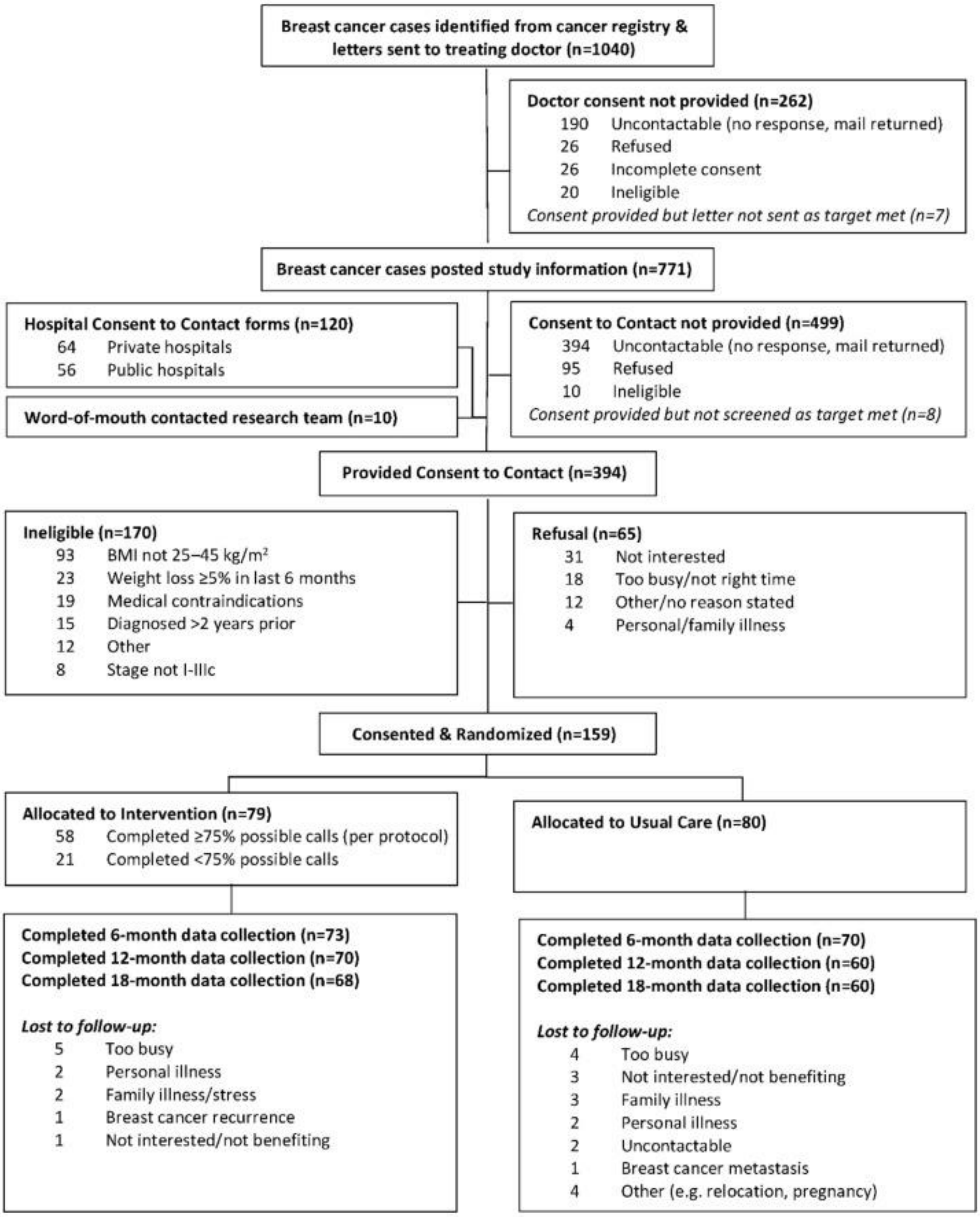

Supplementary Figure 1: The participant flow diagram for the Living Well after Breast Cancer trial [1].

Supplementary Tables

Supplementary Table 1: Baseline characteristics of Living Well after Breast Cancer study participants in the intervention (n=79) and usual care groups (n=80) recruited from Brisbane, Australia; data collected between 2012-2016. [a]

Characteristics	All (n=159)	Intervention (n=79)	Usual care (n=80)
Sociodemographic	←mean ± standard deviation→		
Age at study baseline (years)	55.4 ± 9.2	55.9 ± 9.1	54.9 ± 9.3
BMI (kg/m^2)	31.4 ± 5.0	31.4 ± 4.9	31.3 ± 5.2
	←n (%)→		
**Highest education level** [b]			
High school or less	64 (40)	32 (41)	32 (40)
Trade/technical	37 (23)	16 (20)	21 (26)
University or higher	58 (37)	31 (39)	27 (34)
**Employment status** [c]			
Full-time, part-time, or casual	94 (59)	50 (63)	44 (55)
Retired	34 (21)	19 (24)	15 (19)
Other	31 (20)	10 (13)	21 (26)
**Gross household income** (AUD) [d,e]			
<$1578 weekly	71 (44)	37 (47)	34 (43)
≥$1578 weekly	71 (44)	33 (42)	38 (47)
Not known/reported	17 (11)	9 (11)	8 (10)
Married or living together	110 (69)	54 (68)	56 (70)
Ethnicity (Caucasian)	156 (98)	78 (99)	78 (97)
**Charlson Comorbidity Index**			
0	98 (62)	47 (60)	51 (64)
1	31 (19)	13 (16)	18 (22)
≥2	30 (19)	19 (24)	11 (14)
Breast cancer & treatment-related	←median [IQR]→		
Time since diagnosis (months)	9.5 [5.5]	9.6 [4.4]	9.5 [5.3]
	←n (%)→		
**Menopausal status at diagnosis**			
Pre-menopausal	59 (37)	28 (35)	31 (39)
Peri-menopausal	21 (13)	6 (8)	15 (19)
Post-menopausal	79 (50)	45 (57)	34 (42)

Characteristics	All (n=159)	Intervention (n=79)	Usual care (n=80)
**Tumour stage** [f]			
I	86 (55)	40 (51)	46 (58)
II	54 (34)	30 (38)	24 (30)
III	18 (11)	9 (11)	9 (11)
**Tumor receptor status** [g]			
ER/PR+ /ER+ only/ PR+ only	139 (89)	67 (86)	72 (91)
Both negative	18 (11)	11 (14)	7 (9)
**Type of surgery** [f]			
Breast conserving	98 (62)	49 (62)	49 (62)
Mastectomy	60 (38)	30 (38)	30 (38)
**Breast cancer treatments**			
Surgery only	10 (6)	4 (5)	6 (7)
Surgery and chemotherapy	23 (14)	12 (15)	11 (14)
Surgery and radiation	50 (32)	27 (34)	23 (29)
Surgery, chemotherapy, and radiation	76 (48)	36 (46)	40 (50)

Abbreviations: AUD = Australian dollar; BMI = body mass index; ER = estrogen receptor; PR = progesterone receptor.

[a] Data are number and percentage of participants n (%), mean ± SD, or median [IQR]. [b] For highest level of education received, data were collected as: Completed primary school; High school Grade 10; High school Grade 12; Trade/technical certificate or diploma; Undergraduate university degree; Postgraduate diploma; Master degree or doctorate, and collapsed as above. [c] For employment status, data were collected as: Full time paid work; Part time paid work; Causal paid work; Home duties and not looking for work; Retired; Unable to work; Unemployed – looking for work; Student; Other, and collapsed as above. [d] For gross household income, data were collected as: ≤$540 per week /≤$28,080 per year; $541-1,007 per week/ $28,081-52,364 per year; $1,008-1,577 per week/$52,365-82,004 per year; $1,578-2,390 per week/$82,005-124,280 per year; ≥$2,391 per week; I don't know; Unknown before tax/but known after tax; I don't want to answer this question, and collapsed as above. [e] Threshold indicates top 40% / bottom 60% of Australian population household incomes. [f] n=158; one participant missing from the usual care group. [g] n=156; two participants missing one from the usual care and one from the intervention group.

Supplementary Table 2: Definitions and categories of eating frequency, timing of meals, and sleep parameters.

Eating and sleep variables	Definition	Categories
Eating frequency [a]	Estimated as any eating and drinking episode except water at a single timepoint over a 24-hour period [2]. [b]<br><br>Categorised based on our sample's median eating frequency due to inconsistencies in how eating frequency is measured and reported in the literature.<br><br>Evidence from non-cancer populations suggest that eating more frequently is associated with lower CRP [3] and lower hazard ratio for cardiovascular disease (CVD) mortality (≥6 versus 4 times/day) [4].<br><br>An alternative definition of eating frequency has also been used frequently in the literature, i.e., an individual eating occasion (EO) must contain a minimum energy content of 210 kJ and is separated in time from the preceding and succeeding EO by >15 min [5]. Application of this definition demonstrated that the prevalence of eating frequency was similar for both the breast cancer sample and NNPAS sample. When comparing the two definitions, the first (criteria: ≥1 kJ) was more sensitive to the snacking behaviours (snacks <210 kJ) in our sample of breast cancer survivors.	≤6 and >6 times a day
Proportion of daily calories consumed after 5 PM [a]	Estimated by dividing total calories consumed after 5 PM by total daily calories and multiplying by 100 [3]. [b]<br><br>Categorised based on existing evidence that suggests fasting for longer than 13 hours per night is associated with lower CRP, among women who ate less than 30% of their total daily calories after 5 PM [3]. Additionally, in a sample of women without a breast cancer diagnosis, participants that self-reported ≥33% (versus <33%) energy intake after 5 PM were more likely to be overweight/obese [6].<br><br>Evidence from non-cancer populations also suggest that greater proportion of calories consumed after 5 PM is associated with higher CRP [3].	<30% and ≥30% of daily calories consumed after 5 PM
Eating after 8 PM [a]	Participants were considered to eat after 8 PM if they consumed food or drinks of 105 kJ/25 kcal or more [7]. [b]	Yes or No

Eating and sleep variables	Definition	Categories
Nightly fasting duration [a]	Estimated by calculating the time between the first and last eating episode within the 24-hour recall period and subtracting this time interval from 24 [7]. [b]<br><br>Categorised based on existing evidence between nightly fasting duration and breast cancer recurrence [7].	<13 hours and ≥13 hours per night
Sleep duration [c]	Sleep duration (in hours) per night was calculated as the difference between reported sleep time on a particular day and wake time on the subsequent day and categorised consistent with the National Sleep Foundation's sleep recommendation for adults (i.e., 7-9h per night) [8].<br><br>Estimated sleep duration was weighted to account for weekdays and weekend days ([mean sleep duration $(\text{hours})_{weekday} \times 5 +$ mean sleep duration $(\text{hours})_{weekend} \times 2)/7$]).<br><br>Three women did not report sleep on weekends, so sleep duration was estimated from the average of sleep on weekdays only.	<7 hours (h), ≥7h to ≤9h, >9h of sleep per night
Sleep quality	Sleep quality was estimated by the following single item questions from the ISI questionnaire [9].<br><br>1) "Please rate the severity of any of the following sleep problem(s) over the past 2 weeks" – Difficulty falling asleep; Difficulty staying asleep; Problem waking up too early (5-point Likert scale none to very severe).<br><br>2) "How satisfied / dissatisfied are you with your current sleep pattern?" (5-point Likert scale very satisfied to very dissatisfied).	1) None, mild, moderate, severe/very severe<br><br>2) Very satisfied, satisfied, moderately satisfied, dissatisfied/very dissatisfied.
Chronotype	Estimated by the midpoint of sleep on weekends, corrected for sleep debt accumulated over weekdays (MSF_{sc}) [10].	Early, intermediate, and late based on tertile cut-offs.

Eating and sleep variables	Definition	Categories
	Using daily sleep midpoints:<br>MSF (mid-point of sleep on weekend) = sleep onset on weekend + (sleep duration on weekend/2)<br><br>If sleep duration on weekend > weekday, MSFsc (corrected for sleep debt) = MSF – (sleep duration on weekend – sleep duration on weekday)/2<br><br>Else, MSFsc = MSF<br><br>Chronotype estimated via MSF_{sc} is a continuous trait, dependent on individual (age, sex) and contextual factors (location, culture) [11]. However tertile cut-offs, for early, intermediate, and late were used as done previously [11-13].	
Eating behaviours estimated from the 2011-2012 National Nutrition and Physical Activity Survey	Prevalence of eating behaviours were estimated from a single 24-hour dietary recall. All percentage estimates from NNPAS data account for person weightings and survey design [14].	All eating behaviours were categorised based on definitions provided above.
Sleep duration estimated from the 2011-2012 National Nutrition and Physical Activity Survey	Sleep duration (in hours) on the night before the survey was calculated as time between going to bed (i.e., the light was turned off with intention to sleep) and waking up (for the last time) [15].	<7 h, ≥7h to ≤9h, >9h of sleep per night

[a] Eating frequency and timing of meals were defined using a small number of available studies. Prevalence estimates using these cut-offs are therefore exploratory. Across the literature, there is heterogeneity in how eating frequency and timing are defined. For this study, definitions were based on evidence examining the associations of frequency and/or timing of meals with prognostic factors and breast cancer outcomes. [b] Behaviours were estimated using the average of two non-consecutive 24-hour dietary recalls. [c] Sleep duration was estimated by the mean sleep reported in the sleep logs across all reported days, and weighted for weekdays and weekends. Women were not required to complete sleep logs for all seven days to be included in these analyses. At baseline the majority of our sample recorded sleep for all seven days (n=141; 89%), with only fifteen women reporting sleep for six days (9%) and three reporting for five days (2%).

Supplementary Table 3: Comparison of eating frequency, timing of meals, and sleep duration, stratified by age, between breast cancer survivors from the Living Well after Breast Cancer trial and women from an Australian national survey of similar age- and BMI-range. [a]

		<55 years				≥55 years			
		Breast cancer survivors (n=87)		NNPAS (n=942)		Breast cancer survivors (n=72)		NNPAS (n=793)	
Eating frequency and timing of meals	Categories	n	% (95% CI)	n	% (95% CI)	n	% (95% CI)	n	% (95% CI)
Eating frequency (times/day)	≤6	39	45 (34-56)	594	63 (60-66)	40	56 (43-67)	458	58 (54-61)
	>6	48	56 (44-66)	348	37 (34-40)	32	44 (33-57)	335	42 (39-46)
Proportion of calories after 5PM (%)	≥30	70	80 (70-88)	754	80 (77-82)	59	82 (71-90)	617	78 (75-81)
	<30	17	19 (12-29)	188	20 (17-23)	13	18 (10-29)	176	22 (19-25)
Eating after 8PM	Yes	67	77 (67-85)	518	55 (52-58)	40	55 (43-67)	389	49 (45-52)
	No	20	23 (15-33)	424	45 (42-48)	32	45 (33-57)	404	51 (47-54)
Nightly fasting duration (hours)	<13	73	84 (74-91)	708	75 (72-78)	59	82 (71-90)	591	75 (71-77)
	≥13	14	16 (9-25)	234	25 (22-28)	13	18 (9-29)	202	25 (22-29)
Sleep duration (hours)	<7	4	5 (1-11)	195	21 (18-23)	1	1 (0-7)	135	17 (14-20)
	≥7 to ≤9	59	68 (57-77)	603	64 (61-67)	53	74 (62-83)	499	63 (59-66)
	>9	24	27 (18-38)	144	15 (13-18)	18	25 (15-37)	159	20 (17-23)

Abbreviations: NNPAS = National Nutrition and Physical Activity Survey. [a] Data are number and percentage of participants n (%), including 95% confidence intervals.

Supplementary Table 4: Demographic and clinical subgroups by eating frequency, timing of meals, and sleep duration in breast cancer survivors from the Living Well after Breast Cancer trial.[a]

Characteristics	Eating frequency (times/day)		p	Proportion of daily calories after 5 PM (%)		p	Eating after 8 PM		p	Nightly fasting duration (hours)		p	Sleep duration (hours)		p [b]
	>6 (n=80)	≤6 (n=79)		<30 (n=30)	≥30 (n=129)		No (n=52)	Yes (n=107)		≥13 (n=27)	<13 (n=132)		≥7 to ≤9 (n=112)	>9 (n=42)	
Sociodemographic-related															
←mean ± standard deviation→															
Age at study baseline (years)	54.4 ± 8.5	56.5 ± 9.7	0.15	55.7 ± 8.5	55.3 ± 9.4	0.85	57.4 ± 9.8	54.4 ± 8.7	0.05	55.0 ± 9.8	55.5 ± 9.1	0.82	53.8 ± 5.4	55.1 ± 8.8	0.75
BMI (kg/m^2)	30.5 ± 4.2	32.2 ± 5.6	**0.028**	30.9 ± 4.1	31.4 ± 5.2	0.65	31.2 ± 4.9	31.4 ± 5.1	0.85	31.4 ± 5.3	31.3 ± 4.9	0.94	31.3 ± 3.0	31.6 ± 5.2	0.73
←n (%)→															
**Highest education level**			**0.022**			**0.013**			0.067			0.91			0.93
High school or less	24 (30)	40 (51)		17 (57)	47 (36)		27 (52)	37 (35)		11 (41)	53 (40)		45 (40)	18 (43)	
Trade/technical	20 (25)	17 (21)		9 (30)	28 (22)		12 (23)	25 (23)		7 (26)	30 (23)		27 (24)	9 (21)	
University or higher	36 (45)	22 (28)		4 (13)	54 (42)		13 (25)	45 (42)		9 (33)	49 (37)		40 (36)	15 (36)	
**Employment status**			0.30			0.21			**0.031**			**0.017**			0.38
Full-time, part-time, or casual	52 (65)	42 (53)		14 (47)	80 (62)		24 (46)	70 (65)		10 (37)	84 (64)		20 (18)	11 (26)	
Retired	14 (18)	20 (25)		7 (23)	27 (21)		17 (33)	17 (16)		7 (26)	27 (20)		23 (20)	10 (24)	
Other	14 (17)	17 (22)		9 (30)	22 (17)		11 (21)	20 (19)		10 (37)	21 (16)		69 (62)	21 (50)	
			0.13			0.67			**0.049**			0.24			0.55

Characteristics	Eating frequency (times/day)		p	Proportion of daily calories after 5 PM (%)		p	Eating after 8 PM		p	Nightly fasting duration (hours)		p	Sleep duration (hours)		p [b]
	>6 (n=80)	≤6 (n=79)		<30 (n=30)	≥30 (n=129)		No (n=52)	Yes (n=107)		≥13 (n=27)	<13 (n=132)		≥7 to ≤9 (n=112)	>9 (n=42)	
**Gross Household income** (AUD)															
<$1578 weekly	31 (39)	40 (51)		15 (50)	56 (43)		29 (56)	42 (39)		13 (48)	58 (44)		48 (43)	22 (52)	
≥$1578 weekly	42 (52)	29 (37)		13 (43)	58 (45)		16 (31)	55 (51)		9 (33)	62 (47)		53 (47)	16 (38)	
Not known/reported	7 (9)	10 (13)		2 (7)	15 (12)		7 (13)	10 (9)		5 (19)	12 (9)		11 (10)	4 (10)	
Married or living together	50 (62)	60 (76)	0.066	16 (53)	94 (73)	**0.037**	36 (69)	74 (69)	0.99	19 (70)	91 (69)	0.88	76 (68)	29 (69)	0.89
Breast cancer & treatment-related															
←median [IQR]→															
Time since diagnosis (months)	9.7 [4.9]	9.5 [5.5]	0.97	9.7 [6.0]	9.5 [4.7]	0.92	9.8 [7.2]	9.5 [4.1]	0.67	9.8 [4.9]	9.4 [5.0]	0.39	9.5 [4.8]	9.6 [5.9]	0.55
←n (%)→															
Prognostic stage [c]			0.15			0.23			0.08			0.75			0.35
I	41 (51)	51 (65)		21 (72)	71 (55)		26 (51)	66 (62)		16 (62)	76 (58)		63 (56)	27 (66)	
II	18 (23)	15 (19)		4 (14)	29 (22)		16 (31)	17 (16)		6 (23)	27 (20)		24 (21)	8 (19)	
III	21 (26)	12 (15)		4 (14)	29 (22)		9 (18)	24 (22)		4 (15)	29 (22)		25 (22)	6 (15)	
Chemotherapy treatment	56 (70)	43 (54)	**0.043**	19 (63)	80 (62)	0.89	29 (56)	70 (65)	0.24	16 (59)	83 (63)	0.72	67 (60)	29 (69)	0.29
Radiotherapy treatment	64 (80)	62 (78)	0.81	24 (80)	102 (79)	0.91	43 (83)	83 (78)	0.46	24 (89)	102 (77)	0.17	88 (79)	33 (79)	1.00
←mean ± standard deviation→															
Menopausal Symptoms –	2.3 ± 2.1	2.6 ± 2.1	0.49	2.9 ± 1.9	2.3 ± 2.1	0.16	2.4 ± 2.2	2.5 ± 2.1	0.91	2.9 ± 2.0	2.4 ± 2.1	0.29	2.4 ± 2.1	2.3 ± 2.0	0.66

Characteristics	Eating frequency (times/day)		p	Proportion of daily calories after 5 PM (%)		p	Eating after 8 PM		p	Nightly fasting duration (hours)		p	Sleep duration (hours)		p [b]
	>6 (n=80)	≤6 (n=79)		<30 (n=30)	≥30 (n=129)		No (n=52)	Yes (n=107)		≥13 (n=27)	<13 (n=132)		≥7 to ≤9 (n=112)	>9 (n=42)	
Vasomotor subscale															
								←n (%)→							
**Charlson Comorbidity Index**			0.08			0.82			0.90			0.32			0.35
0	54 (67)	44 (56)		20 (67)	78 (60)		32 (62)	66 (62)		20 (74)	78 (59)		72 (64)	23 (55)	
1	10 (13)	21 (26)		5 (16)	26 (20)		11 (21)	20 (19)		3 (11)	28 (21)		22 (20)	8 (19)	
≥2	16 (20)	14 (18)		5 (16)	25 (19)		9 (17)	21 (20)		4 (15)	26 (20)		18 (16)	11 (26)	

[a] Data are n (%), mean ± SD, or median [IQR]. [b] p-value excludes sleep duration <7 hours due to small sample size. [c] n=158; one participant missing from the usual care group. Descriptive statistics were performed to report on the sociodemographic and clinical correlates of eating frequency, timing of meals, and sleep duration in a sample of breast cancer survivors at the study baseline, using Mann-Whitney tests (where median [IQR] are reported), independent sample t-tests (where mean ±SD are reported), and Chi-squared tests (where n [%] are reported).

Supplementary Table 5: Prevalence of indicators of sleep quality in breast cancer survivors from the Living Well after Breast Cancer trial. [a]

Sleep parameters [b]	All (n=155)
**Difficulty falling asleep**	
None	55 (35)
Mild	45 (29)
Moderate	31 (21)
Severe-Very severe	24 (15)
**Difficulty staying asleep**	
None	32 (21)
Mild	40 (26)
Moderate	43 (28)
Severe-Very severe	40 (26)
**Problem waking up too early**	
None	49 (31)
Mild	32 (21)
Moderate	43 (28)
Severe-Very severe	31 (20)
**How satisfied/dissatisfied are you with your current sleep pattern**	
Very satisfied	14 (9)
Satisfied	39 (25)
Moderately satisfied	50 (32)
Dissatisfied -Very dissatisfied	52 (34)

[a] Data are number and percentage of participants n (%). [b] n=155, four participants did not respond to the questions on self-reported sleep quality.

Supplementary Table 6: Co-occurrence of eating frequency, timing of meals, and sleep duration with indicators of sleep quality in breast cancer survivors from the Living Well after Breast Cancer trial. [a]

Characteristics	Eating frequency (times/day)		p	Proportion of calories after 5 PM (%)		p	Eating after 8 PM		p	Nightly fasting duration (hours)		p	Sleep duration (hours)		p [b]
	>6 (n=80)	≤6 (n=79)		<30 (n=30)	≥30 (n=129)		No (n=52)	Yes (n=107)		≥13 (n=27)	<13 (n=132)		≥7 to ≤9 (n=112)	>9 (n=42)	
**Problem falling asleep** [c]			0.08			0.29			**0.037**			0.12			0.20
None	35 (45)	20 (26)		9 (30)	46 (37)		14 (27)	41 (40)		6 (22)	49 (38)		43 (40)	11 (26)	
Mild	19 (24)	26 (34)		7 (23)	38 (30)		15 (29)	30 (29)		8 (30)	37 (29)		32 (30)	12 (29)	
Moderate	12 (15)	19 (25)		6 (20)	25 (20)		9 (17)	22 (21)		5 (18)	26 (20)		16 (15)	12 (28)	
Severe-Very severe	12 (15)	12 (16)		8 (27)	16 (13)		14 (27)	10 (10)		80 (30)	16 (13)		17 (16)	7 (17)	
**Difficulty staying asleep** [c]			0.25			0.08			0.99			0.50			0.25
None	17 (22)	15 (19)		4 (13)	28 (22)		11 (21)	21 (20)		5 (18)	27 (21)		22 (20)	9 (21)	
Mild	25 (32)	15 (19)		5 (17)	35 (28)		14 (27)	26 (25)		5 (18)	35 (27)		32 (30)	7 (17)	
Moderate	19 (24)	24 (31)		8 (27)	35 (28)		14 (27)	29 (28)		7 (26)	36 (28)		30 (28)	11 (26)	
Severe-Very severe	17 (22)	23 (30)		13 (43)	27 (22)		13 (25)	27 (26)		10 (37)	30 (23)		24 (22)	15 (36)	
**Problem waking up too early** [c]			0.54			0.65			**0.043**			0.62			0.86
None	26 (33)	23 (30)		9 (30)	40 (32)		15 (29)	34 (33)		10 (37)	39 (30)		32 (30)	15 (36)	
Mild	19 (24)	13 (17)		4 (13)	28 (22)		8 (15)	24 (23)		4 (15)	28 (22)		22 (20)	9 (21)	
Moderate	19 (24)	24 (31)		10 (33)	33 (26)		12 (23)	31 (30)		6 (22)	37 (29)		32 (30)	10 (24)	
Severe-Very severe	14 (18)	17 (22)		7 (23)	24 (19)		17 (33)	14 (14)		7 (26)	24 (19)		22 (20)	8 (19)	
**How satisfied are you with your current sleep pattern** [c]			0.66			0.21			0.60			0.27			0.89
Very satisfied	8 (10)	6 (8)		2 (7)	12 (10)		6 (12)	8 (8)		4 (15)	10 (8)		10 (9)	4 (10)	
Satisfied	22 (28)	17 (22)		6 (20)	33 (26)		10 (19)	29 (28)		5 (18)	34 (26)		29 (27)	9 (21)	

Moderately satisfied	25 (32)	25 (32)	7 (23)	43 (34)	17 (33)	33 (32)	6 (22)	44 (34)	33 (31)	15 (36)
Dissatisfied -Very dissatisfied	23 (29)	29 (38)	15 (50)	37 (30)	19 (36)	33 (32)	12 (44)	40 (31)	36 (33)	14 (33)

[a] Data are number and percentage of participants n (%). [b] p-value excludes sleep duration <7 hours due to small sample size. [c] n=155, four participants did not respond to the questions on self-reported sleep quality. Descriptive statistics were performed to report on the co-occurrence of eating frequency, timing of meals, and sleep in a sample of breast cancer survivors at the study baseline, with differences between groups estimated using Chi-squared tests.

References

1. Reeves MM, Terranova CO, Erickson JM, Job JR, Brookes DSK, McCarthy N, et al. Living Well after Breast Cancer Randomized Controlled Trial Protocol: Evaluating a Telephone-Delivered Weight Loss Intervention versus Usual Care in Women Following Treatment for Breast Cancer. BMC Cancer. 2016;16(1):830. https://doi.org/10.1186/s12885-016-2858-0

2. Srour B, Plancoulaine S, Andreeva VA, Fassier P, Julia C, Galan P, et al. Circadian Nutritional Behaviours and Cancer Risk: New Insights from the Nutrinet-Santé Prospective Cohort Study: Disclaimers. Int J Cancer. 2018;143(10):2369-79. https://doi.org/10.1002/ijc.31584

3. Marinac CR, Sears DD, Natarajan L, Gallo LC, Breen CI, Patterson RE. Frequency and Circadian Timing of Eating May Influence Biomarkers of Inflammation and Insulin Resistance Associated with Breast Cancer Risk. PloS one. 2015;10(8):e0136240-e. https://doi.org/10.1371/journal.pone.0136240

4. Chen HJ, Wang Y, Cheskin LJ. Relationship Between Frequency of Eating and Cardiovascular Disease Mortality in U.S. Adults: The NHANES III Follow-Up Study. Ann Epidemiol. 2016; 26(8):527-33. https://doi.org/10.1016/j.annepidem.2016.06.006

5. Leech RM, Worsley A, Timperio A, McNaughton SA. Characterizing Eating Patterns: A Comparison of Eating Occasion Definitions. AJCN. 2015;102(5):1229-37. https://doi.org/10.3945/ajcn.115.114660

6. Wang JB, Patterson RE, Ang A, Emond JA, Shetty N, Arab L. Timing of Energy Intake During the Day is Associated with the Risk of Obesity in Adults. J Hum Nutr Diet.. 2014;27(s2):255-62. https://doi.org/https://doi.org/10.1111/jhn.12141

7. Marinac CR, Nelson SH, Breen CI, Hartman SJ, Natarajan L, Pierce JP, et al. Prolonged Nightly Fasting and Breast Cancer Prognosis. JAMA Oncology. 2016; 2(8):1049-55. https://doi.org/10.1001/jamaoncol.2016.0164

8. Hirshkowitz M, Whiton K, Albert SM, Alessi C, Bruni O, DonCarlos L, et al. National Sleep Foundation's Updated Sleep Duration Recommendations: Final Report. Sleep Health. 2015;1(4):233-43. https://doi.org/10.1016/j.sleh.2015.10.004

9. Veqar Z, Hussain ME. Validity and Reliability of Insomnia Severity Index and its Correlation with Pittsburgh Sleep Quality Index in Poor Sleepers among Indian University Students. IJAMH. 2017;32(1). https://doi.org/10.1515/ijamh-2016-0090

10. O'Loughlin J, Casanova F, Jones SE, Hagenaars SP, Beaumont RN, Freathy RM, et al. Using Mendelian Randomisation Methods to Understand whether Diurnal Preference is Causally Related to Mental Health. Mol Psychiatry. 2021;26(11):6305-16. https://doi.org/10.1038/s41380-021-01157-3

11. Roenneberg T. Having Trouble Typing? What on Earth is Chronotype? Journal of Biological Rhythms. 2015;30(6):487-91. doi: 10.1177/0748730415603835

12. Juda M, Vetter C, Roenneberg T. Chronotype Modulates Sleep Duration, Sleep Quality, and Social Jet Lag in Shift-Workers. J Biol Rhythms. 2013;28(2):141-51. https://doi.org/10.1177/0748730412475042

13. Vetter C, Juda M, Roenneberg T. The Influence of Internal Time, Time Awake, and Sleep Duration on Cognitive Performance in Shiftworkers. Chronobiol Int. 2012;29(8):1127-38. https://doi.org/10.3109/07420528.2012.707999

14. Australian Bureau of Statistics. Australian Health Survey: Users' Guide, 2011–13. 2013. https://www.abs.gov.au/methodologies/australian-health-survey-nutrition-first-results-foods-and-nutrients-methodology/2011-12. Accessed on 9 November 2024.

15. Australian Bureau of Statistics. Sleep 2013. 2013. https://www.abs.gov.au/ausstats/abs@.nsf/Lookup/8E315FB62A0FF8A7CA257BAC001F6DE8?opendocument. Accessed on 9 November 2024.
